# Alloreactive CD154-expressing T-cell subsets with differential sensitivity to the immunosuppressant, belatacept: potential targets of novel belatacept-based regimens

**DOI:** 10.1038/srep15218

**Published:** 2015-10-16

**Authors:** Chethan Ashokkumar, Bishu Ganguly, Robert Townsend, Jaimie White, Samantha Levy, Michael Moritz, George Mazariegos, Qing Sun, Rakesh Sindhi

**Affiliations:** 1 Plexision, Inc., 4424 Penn Avenue, #202, Pittsburgh, PA 15224 USA; 2 Bristol-Myers-Squibb, PO Box 4000, Princeton, NJ 08543-4000 USA; 3 Children’s Hospital of Pittsburgh of University of Pittsburgh Medical Center, 4401 Penn Avenue, Pittsburgh, PA 15224. USA

## Abstract

Belatacept blocks CD28-mediated T-cell costimulation and prevents renal transplant rejection. Understanding T-cell subset sensitivity to belatacept may identify cellular markers for immunosuppression failure to better guide treatment selection. Here, we evaluate the belatacept sensitivity of allo-antigen-specific CD154-expressing-T-cells, whose T-cytotoxic memory (TcM) subset predicts rejection with high sensitivity after non-renal transplantation. The belatacept concentration associated with half-maximal reduction (EC_50_) of CD154 expression was calculated for 36 T-cell subsets defined by combinations of T-helper (Th), Tc, T-memory and CD28 receptors, following allostimulation of peripheral blood leukocytes from 20 normal healthy subjects. Subsets were ranked by median EC_50_, and by whether subset EC_50_ was correlated with and therefore could be represented by the frequency of other subsets. No single subset frequency emerged as the significant correlate of EC_50_ for a given subset. Most (n = 25) T-cell subsets were sensitive to belatacept. Less sensitive subsets demonstrated a memory phenotype and absence of CD28 receptor. Potential drug-resistance markers for future validation include the low frequency highly differentiated, Th-memory-CD28-negative T-cells with the highest median EC_50_, and the least differentiated, high-frequency Tc subset, with the most CD28-negative T-cells, the third highest median EC_50_, and significant correlations with frequencies of the highest number of CD28-negative and memory subsets.

Belatacept (Nulojix^TM^, Bristol-Myers Squibb, Princeton, NJ), a novel immunosuppressant, was recently approved for prophylaxis of renal transplant rejection, but demonstrated numerically higher rejection rates compared with control immunosuppression in one of the pivotal trials^1^. Alternative belatacept-based regimens may improve outcomes if recipients prone to immunosuppression failure or rejection can be identified preemptively. This task requires that a measurable target be identified, which demonstrates poor response to or is resistant to belatacept. Belatacept is a modified human fusion protein in which the extracellular domain of the cytotoxic T-lymphocyte antigen-4 is linked to the Fc fragment of humanized IgG1 (CTLA4-Ig). Belatacept competes with CD28 for binding to B7 on antigen presenting cells, and inhibits T-cell alloresponses by blocking CD28-mediated T-cell co-stimulation^2^. In previous studies, CD28-negative (CD28-) T-cell subsets and memory subsets have shown reduced dependence on CD28-mediated costimulation^3^,^4^. Therefore, variability in the composition of memory and CD28- T-cells within an individual may explain differences in clinical response to belatacept. Consistent with these reports, the proliferative alloresponse of human T-cytotoxic memory cells, which are CD28-, seems relatively resistant to inhibition with belatacept *in vitro*^5^. However, the relative sensitivity of other common T-cell subsets to belatacept and its variation between individuals has not been well-characterized. This information may be useful, because rejection-prone recipients require more immunosuppression to achieve comparable inhibition of the various types of peripheral blood lymphocytes^6^. Localizing cellular resistance to belatacept to ‘finer’ T-cell subsets may identify those which are resistant to belatacept, and help to better direct the selection of adjunctive immunosuppression to enhance efficacy.

Rejection-prone recipients can be identified with high sensitivity using alloantigen-specific T-cytotoxic memory cells, which express the costimulation receptor, CD154 (CD154+TcM)^7^,^8^,^9^,^10^. The disease-specificity of this subset relative to others has been established with logistic regression in recipients of liver, intestine and renal allografts. The test system is approved to predict rejection in children with liver or intestine transplantation (Pleximmune^TM^, Plexision, Inc., Pittsburgh, PA). Whether CD154+TcM or other alloreactive T-cell subsets, e.g., those that do not express CD28, are relatively resistant to belatacept between individuals has not been evaluated. Establishing resistance of the alloresponse to immunosuppressants in clinical patients can be difficult because multiple samples between two consecutive doses of a drug are required to establish effect: concentration relationships. The relative number or the frequency of such resistant subsets could serve as an easily measured substitute for functional measurements of drug resistance. Whether this approach can succeed is not known. Information about the resistance of alloresponsive T-cell subsets to belatacept may help identify recipients at risk for belatacept resistant acute rejection. This report describes the relative *in vitro* sensitivity of alloreactive T-cell subsets to belatacept-mediated inhibition in peripheral blood lymphocytes (PBL) from adult normal healthy volunteers (NHV). Effect: concentration analyses are used to identify candidate subsets, which appear suited for clinical validation from among 36 T-cell subsets. Belatacept-treated transplant recipients were not available to determine whether the candidate subsets identified in this study distinguish rejection-prone recipients on this regimen. We use a pilot experiment with research blood samples from available transplant recipients to perform a preliminary assessment.

## Results

### T-cytotoxic cells have higher content of CD28-negative cells

PBL from 32 normal healthy volunteers (NHV) were phenotyped by flow cytometry to evaluate the distribution of CD28- cells among memory (CD45RO+) and naive (CD45RO−) subsets of four major or parent T-cell subsets. These four parent subsets consist of T (CD3+), Th (CD3 + CD4+), Tc (CD3 + CD8+), and double-negative T-cells (CD3 + CD4-CD8−). The parent subset and its memory and naive, CD28+ and CD28− subsets together made up five subsets for each of the four parent subsets. The memory and naïve subsets were each divided further into CD28+ and CD28- subsets thus contributing four additional subsets for a total of nine subsets for each parent subset. In all, 36 total T-cell subsets were defined by this approach. The flow cytometry gating strategy is shown in Supplementary Figure S1.

The Tc compartment contributes significantly more CD28-negative T-cells compared with Th (8.9 ± 7.9% vs 2.1 ± 4.63%. p = 0.0001) to the overall CD28-negative T-cells (14 ± 12.7%) in the T-cell compartment. Frequencies of CD28-T-cell were not markedly different in the memory or naïve subsets of the Th (1.2 ± 2.4% vs 0.8 ± 2.3%, respectively) or Tc (3.5 ± 3.7% vs 5.2 ± 5.5%, respectively (Table 1).

### Belatacept inhibits alloreactivity of all T-cell subsets

Alloreactivity was measured by CD154 induction on T cells and proliferating T-cells within each subset with flow cytometry.

The frequency of CD154+cells induced within each of 36 T-cell subsets was determined in lymphocyte co-culture. Responder PBL were obtained from 20 NHV. Stimulator PBL were depleted of CD3+ cells using magnetic CD3 MicroBeads according to the manufacturer’s instructions (Miltenyi Biotec, Auburn, CA) and irradiated. Responder PBL were cultured overnight with HLA-mismatched irradiated T-cell-depleted stimulator PBL in 1: 2 ratio with seven belatacept concentrations ranging from 0–100 μg/ml. The culture medium included anti-CD154-phycoerythrin for non-permeabilizing detection of intracellular CD154 as described previously^7^,^8^,^9^,^10^.

To confirm that allostimulation occurred in our experiments, parallel validation assays tested the effect of belatacept on the proliferative alloresponse of all 36 T-cell subsets in four of 20 responders in 7-day co-culture using dilution of the intravital dye carboxyfluoresceinisothocyanate (CFSE, Invitrogen, CA)^11^. In these assays, CFSE^low^T-cells served as the measure of proliferating T-cells in each subset.

**Figure 1:**
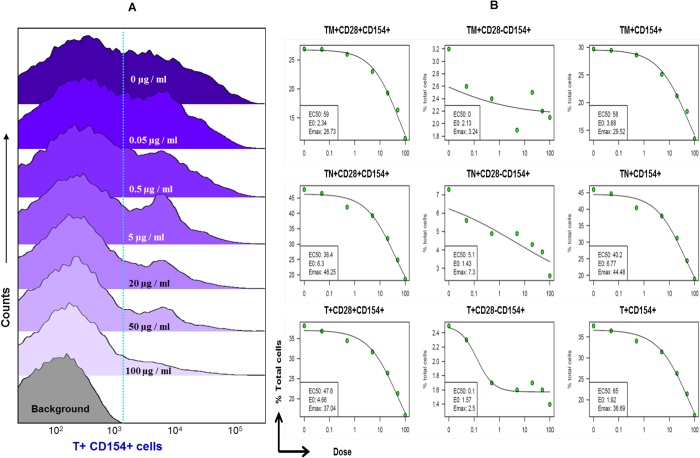
(**A**) Histogram showing the effect of belatacept on CD154+T-cells. Figure 1B. The effect of belatacept concentrations on nine CD154+T-cell subsets is modeled with best-fit four-parameter log-logistic function. Insets show calculated EC_50_. Similar analyses have been performed for nine subsets of each of the Th, Tc and double negative (Dn, CD3+CD4−CD8−) T-cells.

Alloreactivity decreased with increasing belatacept concentrations in all T-cell subsets (Fig. 1A, Supplementary Figure S2). As an established technique, the proliferative alloresponse confirmed that allostimulation and its inhibition with belatacept occurred in our co-culture experiments. Proliferation data were not analyzed further.

### Alloreactive T-cell subsets display variable sensitivity to belatacept

Belatacept sensitivity was estimated with EC_50_, the belatacept concentration measured in μg/ml associated with half-maximal reduction of CD154+cells within each subset in each responder. EC_50_ was calculated with best-fit four-parameter log-logistic function under a Poisson assumption as described in the methods below.

**Figure 2:**
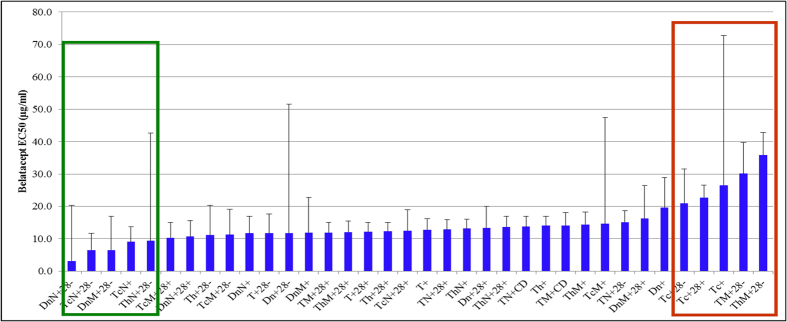
T-cell subsets ranked by increasing (median±SEM) EC_50_. Red and green boxes identify subsets with the highest (>20 μg/ml) and lowest (<10 μg/ml) EC_50_ values, respectively. T = T-cell, Tc = T-cytotoxic cell, Th = T-helper cell, M = memory, N = naïve, Dn=double negative T-cell (CD3 + CD4-CD8-).

Alloreactivity measurements were obtained from 36 T-cell subsets from 20 responders for a total of 720 subsets. The EC_50_ was successfully computed in 676 of 720 total CD154+T-cell subsets (93.9%), with 44 computational failures (6.1%) (Fig. 1B). The EC_50_ varied widely (0–823 μg/ml). For the majority of subsets, EC_50_ values did not follow a normal distribution (Table 2). Therefore, belatacept sensitivity was summarized as median EC_50_, and ranged from 10.35 to 19.7 μg/ml for 25 of 36 CD154+T-cell subsets (Fig. 2). The six most sensitive subsets demonstrated EC_50_ values below 10 μg/ml. The five least sensitive subsets showed values above 20 μg/ml. Among the least sensitive subsets, the ThMCD28- subset required the most belatacept (median 35.8 μg/ml) for half-maximal reduction of ThMCD28- cells, which expressed CD154.

### The relationship between belatacept sensitivity and subset frequency

**Figure 3:**
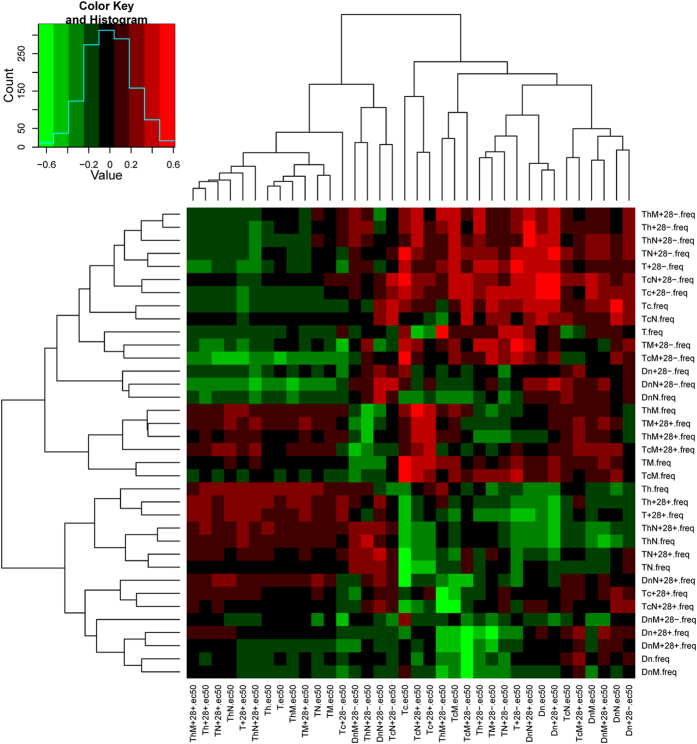
Heatmap from hierarchical clustering analysis shows Spearman correlations between EC_50_ values for each CD154+T-cell subset with frequencies of each of 36 T-cell subsets for 20 responders. Spearman rho values range from −0.6 (green) to 0.6 (red). Tc EC_50_ is correlated with frequencies of several T-cell subsets. ThM28- frequencies are correlated with EC_50_ of several T-cell subsets. T = T-cell, Tc = T-cytotoxic cell, Th = T-helper cell, M = memory, N = naïve, Dn = double negative T-cell (CD3 + CD4-CD8-).

Whether the frequency of a T-cell subset, which is easier to measure than and may serve as a substitute for subset EC_50_, may also identify the rejection-prone recipient with a belatacept-containing regimen can only be established in clinical studies. In this *in vitro* study, it was only possible to determine whether the EC_50_ of a subset was related to the frequency of the same or other T-cell subsets. To this end, multiple correlations were performed between the EC_50_ of a subset with the frequency of all subsets (Supplementary Table S1, Fig. 3).

The EC_50_ of T-cells which did not express CD28 (TCD28-) demonstrated a significant correlation with its frequency (Spearman r = 0.511, p = 0.021), and the frequency of several other subsets. The EC_50_ of every other T-cell subset also showed correlations with the frequencies of several other subsets other than its own frequency. Some of these correlations achieved statistical significance. Interestingly, the EC_50_ of the least belatacept-sensitive ThMCD28- subset showed correlations with the frequencies of 19 of 36 subsets. However, none of these correlations were statistically significant.

Next, the multiple correlations between EC_50_ and frequencies were used to rank subsets further using hierarchical clustering. Among EC_50_-based clusters, the Tc, which has the third highest ranked median EC_50_ (26 μg/ml) is located atop a hierarchy based on correlations with frequencies of the most number (n = 21) of subsets. That this subset also demonstrated the highest ranked mean EC_50_ (86.6 ng/ml) was not used for ranking, because like other subsets, the EC_50_ of Tc did not follow normal distribution in the 20 responders. Significant positive correlations are seen between Tc EC_50_ and the frequencies of subsets which have the memory or the CD28- phenotype such as TM, TcM, TcM28- and TcN28- subsets (p ≤ 0.05). In contrast, Tc EC_50_ has significant negative correlations with the frequencies of subsets, which have the naïve or CD28+ phenotype such as the TN, TN28+, TcN28+, ThN and ThN28+ subsets. These correlations suggest that CD28- and memory subsets of the Tc contribute to or mirror the belatacept resistance of Tc, a finding consistent with the previously reported costimulation-blockade-resistance of memory and CD28- subsets (Supplementary Table S1)^3^,^4^.

### Capturing individual variation with candidate subsets

The abovementioned analyses identified two potential candidate T-cell subsets suited for future clinical validation as markers of individual drug sensitivity, the ThMCD28- and the Tc subsets. Although ThMCD28- frequencies demonstrated greater variation between individuals compared with Tc (coefficient of variation or %CV 193% vs 29.4%, respectively), the EC_50_ of Tc showed greater variation between individuals compared with EC_50_ of ThMCD28- cells (%CV 238% vs 83.5%, Table 3).

### Preliminary assessment of candidate subsets in transplant recipients

To measure the donor-specific alloresponse for clinical rejection-risk assessment, the test system described here is expanded to include two simulators, donor and HLA-non-identical or reference cells^7^,^8^,^9^. The results of these two reactions are expressed as a ratio termed the immunoreactivity index, or IR. If donor-specific CD154+T-cytotoxic memory cells exceed those induced by stimulation with an HLA-non-identical stimulator cell or reference cell, the IR exceeds 1 and the individual is at increased risk of rejection. If the ratio is <1, the recipient is at decreased risk. If donor cells are not available, the test can be performed with ‘surrogate’ donor cells, which are obtained from normal human subjects and are matched at one antigen each at the HLA-A, -B, and -DR loci. Rejection-risk assessment is not compromised with this approach^9^.

**Figure 4:**
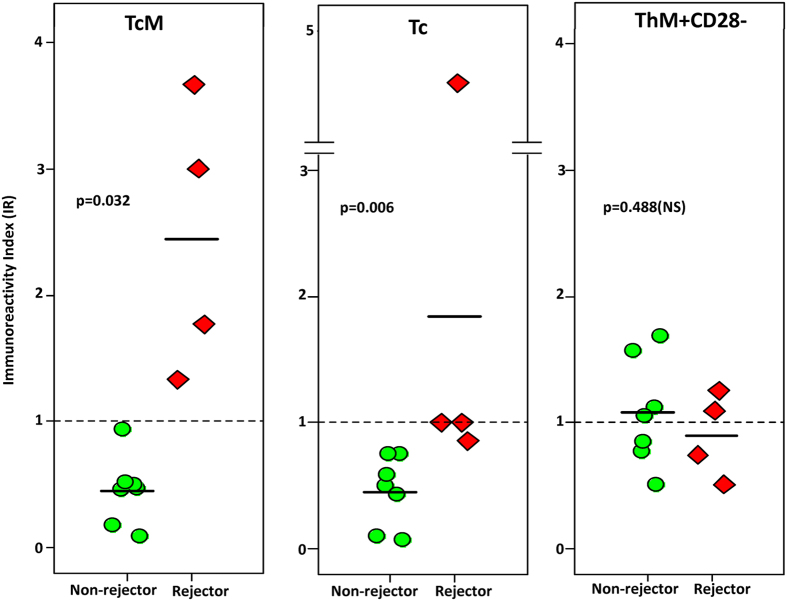
Plots showing mean Immunoreactivity Index (IR) values for T-cytotoxic Memory (TcM), T- cytotoxic (Tc) and T-helper CD28 negative (Th + 28−) cells which express CD154 in blood samples from 11 children who have received liver or intestine transplantation. For each subset, mean values are shown for rejectors (n = 4) and non-rejectors (n = 7). Significantly greater IR values are seen in rejectors compared with non rejectors for the TcM and Tc subsets but not the ThM + 28− subset. Subjects received tacrolimus based immunosuppression and not belatacept.

The donor-specific alloresponse of 36 T-cell subsets was measured and expressed as the IR for each of 36 T-cell subsets using research samples from 11 liver or intestine transplant recipients. The mean age of these recipients at sampling was 9.3 ± 8.2 years (range 1–29 years), and distribution of male: female gender was 7: 4 and race distribution Caucasian: non-Caucasian was 10: 1. All recipients received tacrolimus for immunosuppression. The transplanted allografts consisted of the liver in eight, combined liver-kidney in one, combined liver-intestine in one, and intestine in one subject. Four of eleven recipients experienced acute cellular rejection on the day of sampling (rejectors). Rejection was diagnosed with standard biopsy criteria described previously^12^. The remaining seven recipients were non-rejectors based on clinical information (n = 5) or biopsy (n = 2), on the day of sampling and for the 60-day post-sampling time period^7^. The general demographics and characteristics of these subjects were not different between rejectors and non-rejectors (Table 4). Immunoreactivity indices for all subsets are tabulated in Supplementary Table S2. These data show that the current standard, allospecific CD154+T-cytotoxic memory cells demonstrate IR values >1 in all rejectors and <1 in all non-rejectors and no overlap of IR values between groups (Fig. 4). Further, the mean IR values are significantly greater for rejectors compared with non-rejectors. All of the six other T-cell subsets that demonstrate significantly higher IR values in rejectors compared with non-rejectors are also derived from the CD8 compartment (Supplementary Figure S3). These subsets also include CD154+Tc, which had demonstrated the third-highest EC_50_ in our *in vitro* experiments. The IR values from rejectors and non-rejectors for this subset also showed no overlap (Fig. 4). The IR of the remaining T-cell subsets including ThM+CD28- cells did not differ significantly between rejectors and non-rejectors (Supplementary Figure S3 and Supplementary Table S2). The ThM+CD28- subset had displayed the highest EC_50_ values in our *in vitro* experiments.

## Discussion

Markers of drug response are necessary in order to select the best-suited drug regimen for each individual. Because acute cellular rejection is a sign of immunosuppression failure, localizing this failure to cell subsets, which are relatively insensitive to an immunosuppressant may guide the selection of adjunctive drugs to improve efficacy. A collateral benefit maybe that such markers of failure may also identify recipients at risk for rejection with a particular regimen. We explore this possibility by searching for belatacept-resistant cell targets among T-cell subsets, one of which is being used clinically to predict rejection after liver or intestine transplantation^7^,^8^,^9^,^10^. We demonstrate for the first time that the belatacept sensitivity of the T-cell alloresponse varies widely amongst the 36 T-cell subsets. This finding expands on previous studies, which show that mitogen stimulated expression of cytokines and cell-surface receptors on various peripheral lymphocyte subsets vary widely in their susceptibility to immunosuppressants^6^,^13^,^14^. Using alloantigen-specific T-cell responses also overcomes the inconsistencies of mitogen-stimulated lymphocyte responses in predicting rejection, especially after lymphocyte depleting antibodies, which may induce anergy, and which are increasingly used for transplant immunosuppression^15^,^16^.

Expressed as EC_50,_ or the concentration at which CD154+cells within a given subset are reduced by half, the measurement of belatacept sensitivity shows that the less sensitive subsets either lack CD28, or express the memory marker. These attributes characterize the ThM+CD28- subset, which displays the highest median EC_50_. This finding is also consistent with previous studies in which memory and CD28- subsets of Th have shown resistance to CD28-mediated costimulation-blockade during T-cell receptor stimulation^3^,^4^. For this observation to be clinically relevant, the rejection-prone recipient receiving belatacept would also have to demonstrate lower belatacept sensitivity of donor-specific ThM+CD28- cells compared with other subsets, or perhaps higher absolute frequencies of ThM+CD28- cells. One application of such a clinically validated finding may be that such a rejection-prone recipient may benefit from the addition of drugs, which may have potentially greater effect on Th cells, to a belatacept-containing regimen. T-cell depleting agents cause protracted depletion of Th cells in transplant recipients and exemplify one such anti-rejection agent^17^,^18^. Practical limitations to using ThM+CD28- subset as a potential biomarker of drug resistance include the low frequency of this subset, which may preclude reliable measurements of functional responses. In 32 NHV, including the 20 used as responders in this study, median frequency of ThM + 28- cells among T-cells was 0.17% (range 0.02–9.9) and mean ± SD frequency was 1.24 ± 2.39%.

Subset frequencies are easily measured compared with functional alloresponses, and are therefore attractive as markers of drug sensitivity in the clinical setting. Thus, an important question raised by our findings is whether the relative numbers of one or the other ‘resistant’ subset may distinguish the rejection-prone recipient receiving belatacept, who might therefore require adjunctive immunosuppression, from one who is rejection-free. This question can only be addressed definitively in the clinical setting where the subset frequency or its EC_50_ is evaluated for its relationship to the endpoint of rejection. Outside of such a clinical setting, the frequency of such subset(s) would be expected to have a correlation with and therefore reflect the EC_50_ of a ‘resistant’ subset. The multiple correlations between EC_50_ and subset frequencies, and their additional use for ranking by hierarchical clusters, provide some clues. The EC_50_ of a given T-cell subset demonstrated correlations with the frequencies of several other subsets, suggesting that a subset may influence the EC_50_ of many others, or that multiple subsets may influence the EC_50_ of a particular subset. This is to be expected, because the behaviour of any given CD154-expressing subset is also modulated by known and unknown anti-inflammatory or regulatory mechanisms emanating from other T-cells^19^. In our previous work, allospecific CD154+TcM were inversely correlated with the anti-inflammatory CTLA4-expressing TcM induced simultaneously by the same allostimulus^7^. Interestingly, although many correlations are found between the frequencies of several T-cell subsets and the EC_50_ of ThM+CD28- cells, which displayed the highest median EC_50_, none achieved statistical significance. The TCD28- subset, whose EC_50_ was significantly correlated with its frequency (Spearman r = 0.511, p = 0.021), also showed significant correlations with several other subsets, suggesting multiple influences on subset sensitivity. This potential dependence of subset sensitivity on the frequency of several other subsets makes frequency measurements less suited than cell function to infer relative drug sensitivity in a given individual.

These multiple relationships between EC_50_ and frequencies can also be used to identify alternative subsets for further clinical validation. The belatacept sensitivity of T-cytotoxic cells (Tc), or its EC_50_, was significantly correlated with the frequencies of the most number of T-cell subsets. The positively correlated frequencies were predominantly those of memory and CD28- T-cell subsets, suggesting that these subsets contribute to the relatively high EC_50_, which ranked this subset third among the five least sensitive subsets. Conversely, significant negative correlations were demonstrated between the EC_50_ of Tc and the frequencies of several naïve and CD28+ subsets, raising the possibility that these latter subsets negated to some extent the contribution of the more ‘resistant’ subsets to the overall belatacept sensitivity of Tc. This inference is plausible because the majority of CD28-negative-T-cells are present among Tc (Table 1). Why un-fractionated memory subsets of Tc also contribute to or show reduced sensitivity to belatacept is also explained in part by our choice of CD154 induction as a measure of allostimulation. In experimental models, Tc from allosensitized hosts have shown resistance to costimulation blockade with anti-CD154 antibody when re-challenged with alloantigen, presumably because of acquisition of antigen-specific memory^20^. These observations suggest that the activated CD40-CD40 ligand pathway evidenced by the expression of CD154 in Tc-memory cells (CD154+TcM) may be a marker of a resistant T-cell population. Previous observations further support the contribution of the Tc and its memory subset to immunosuppressant failure. T-cytotoxic cells are resistant to CD28-mediated costimulation blockade in models of hepatocyte allograft rejection^21^. Transplant tolerance cannot be induced in primate models if T-cytotoxic memory cells (TcM) are not ablated^22^. Finally, CD154+TcM predict liver, intestine and kidney allograft rejection, a disease state in which T-cells and B-cells demonstrate two- to four-fold higher EC_50_^7^,^8^,^9^,^10^,^6^.

Whether frequencies of the most resistant ThM+CD28− subset which is a terminally differentiated subset by virtue of the T-memory and CD28 classifiers, or the EC_50_ of Tc, which represent the least differentiated T-cell subset, and contain the most CD28- T-cells provide the greatest dynamic range to identify individuals at higher risk for belatacept-resistant rejection must be resolved through clinical testing. Recent evidence from an intact animal model would seem to favor the CD28-negative Tc or its subsets, which also resist drug-induced apoptosis in humans, as markers of belatacept-resistance during rejection^23^,^24^. This speculation finds some support in our preliminary investigation of the test system in 11 recipients of liver or intestine transplantation. In this investigation, the IR of allospecific T-cytotoxic memory cells, which is approved for clinical use and has met several benchmarks for reproducibility serves as a reference by which to test the performance of the two candidate subsets. Specifically, IR values for this subset showed no overlap between outcome groups, and were significantly higher in rejectors compared with non-rejectors (Fig. 4 and Supplementary Figure S3 top panel). The IR of T-cytotoxic cells similarly shows no overlap in IR values between groups, and was significantly higher in rejectors compared with non-rejectors. The IR of the ThM+CD28- cell, and of all other remaining subsets was not significantly different between rejectors and non-rejectors (Supplementary Figure S3, Supplementary Table S2). These results are at best preliminary but prove that donor-specific alloresponses of many T-cell subsets can be used for clinical rejection-risk assessment. Further, none of the 11 recipients were treated with belatacept. Therefore performance testing of the ThM+CD28- cell, which demonstrated the greatest resistance to belatacept must await additional clinical trials.

In summary, alloreactive T-cell subsets, which express the inflammatory costimulator, CD154, demonstrate wide-ranging susceptibility to CD28-costimulation blockade with belatacept. The less sensitive subsets in our study are characterized by the presence of the memory marker, and the absence of CD28 and appear to be distributed in both the Th and Tc compartment. These subsets are suited for clinical validation as markers of relative belatacept resistance, because immunosuppression failure, which can cause transplant rejection has been associated with resistant alloreactive T-cell subsets previously^6^. During the clinical validation phase, the relative merits of resistant subsets in the T-helper and T-cytotoxic cell compartments must also include assessment of available cell counts and the dynamic range of a candidate subset to permit reliable measurements for clinical decision-making.

## Methods

The methods were carried out in accordance with the approved guidelines of the Chesapeake Institutional Review Board (PRO#6774).

After informed consent under IRB-approved protocol peripheral blood samples from 32 NHV and 11 transplant recipients were used to obtain peripheral blood leukocytes (PBL) by Ficoll density gradient centrifugation (Lympholyte-H, CederlaneLabs Burlington, NC). Samples from 20 of 32 NHV were used for alloreactivity experiments. After T-cell depletion of stimulator cells, CD3+T-cells made up <2% of the remaining CD45+PBL, while cells with known antigen presenting function such as CD19+B-cells and CD14+monocytes were enriched by 4–5 fold (Supplementary Figure S4).

Alloreactivity was measured by the frequency of CD154+cells induced within each T-cell subset in lymphocyte co-culture with responder and irradiated stimulators in a 1:2 ratio, using flow cytometry and previously described procedures^7^,^11^. Alloresponse was measured with the T cell activation marker, CD154, in 36 T-cell subsets. All fluorochrome-labeled antibodies were obtained from BD Biosciences (San Jose, CA).

The proliferative alloresponse was measured with dye dilution of CFSE (carboxyfluoresceinisothiocyanate, Invitrogen, CA) in responder cells which had been prelabeled with CFSE prior to co-culture with stimulator cells, as described previously^11^. Flow cytometry was performed using BD FACSCANTOII flow cytometer, and the FACS-DIVA software.

### Statistical Analysis

Summary descriptive statistics including Kolmogorov-Smirnov and Shapiro-Wilk tests of normality were performed by using SPSS, version 22.0 (SPSS, IBM, Chicago, IL, USA). Belatacept sensitivity was estimated with EC_50_, the belatacept concentration associated with half-maximal reduction of CD154+cells within each subset in each responder. EC_50_ was calculated with best-fit four-parameter log-logistic function under a Poisson assumption:
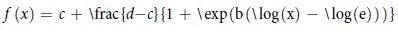
where b = slope, c = lower limit, d = upper limit, and e = EC_50_

The calculations of EC_50_ and hierarchical clustering analysis were performed by using R, version 3.1.0.

## Additional Information

**How to cite this article**: Ashokkumar, C. *et al.* Alloreactive CD154-expressing T-cell subsets with differential sensitivity to the immunosuppressant, belatacept: potential targets of novel belatacept-based regimens. *Sci. Rep.* **5**, 15218; doi: 10.1038/srep15218 (2015).

## Supplementary Material

Supplementary Table S1

Supplementary Information

## Figures and Tables

**Table 1:** Summary frequencies for each of 36 T-cell subsets from 32 normal human volunteers. Frequency of each subset is expressed as the proportion of total T-cell population.

**T-cell Subsets**	**Mean**	**SEM**	**Median**	**SD**	**Min**	**Max**
T	36.5	2.1	36.9	11.6	10.4	54.6
TM	41.9	1.9	39.8	10.6	22.2	64.9
TM+28+	37.0	1.8	35.4	10.5	19.6	62.0
TM+28-	4.9	1.1	2.8	6.0	0.4	25.0
TN	58.6	1.9	60.6	10.5	35.8	78.3
TN+28+	49.7	2.4	50.0	13.4	31.4	76.4
TN+28−	9.0	1.4	5.8	8.1	0.8	27.6
T+28+	86.0	2.2	92.4	12.7	54.4	98.9
T+28−	14.1	2.2	7.6	12.7	1.2	45.8
Th	63.7	1.9	62.9	10.7	43.4	90.5
ThM	30.8	1.5	29.3	8.3	16.3	49.5
ThM+28+	29.6	1.5	28.4	8.4	16.0	49.4
ThM+28−	1.2	0.4	0.2	2.4	0.0	9.9
ThN	33.2	1.8	31.4	10.4	14.1	61.5
ThN+28+	32.4	1.9	30.6	10.8	14.1	60.9
ThN+28−	0.8	0.4	0.0	2.3	0.0	12.6
Th+28+	61.6	2.0	61.0	11.4	41.5	90.5
Th+28−	2.1	0.8	0.2	4.6	0.0	21.9
Tc	29.1	1.5	30.0	8.5	7.4	45.9
TcM	10.9	0.8	10.8	4.6	3.1	21.0
TcM+28+	7.3	0.6	7.4	3.3	2.5	13.2
TcM+28−	3.5	0.7	2.1	3.7	0.2	14.7
TcN	18.3	1.3	18.3	7.1	3.4	31.5
TcN+28+	13.1	1.2	11.7	6.5	2.6	30.0
TcN+28−	5.2	1.0	2.9	5.5	0.3	20.8
Tc+28+	20.2	1.3	20.9	7.4	5.1	38.0
Tc+28−	8.9	1.4	4.9	7.9	0.5	27.3
Dn	6.0	0.8	4.5	4.8	1.1	21.5
DnM	4.1	0.8	2.6	4.6	0.5	20.3
DnM+28+	2.3	0.3	2.0	1.8	0.4	8.4
DnM+28−	1.8	0.8	0.5	4.3	0.2	19.6
DnN	1.8	0.2	1.5	1.1	0.4	5.0
DnN+28+	1.0	0.1	1.0	0.6	0.2	2.8
DnN+28−	0.8	0.2	0.6	0.9	0.1	4.6
Dn+28+	3.3	0.4	2.9	2.1	0.6	9.4
Dn+28−	2.7	0.8	1.3	4.5	0.5	20.3

^*^The frequency of T-cells is expressed as a proportion of all lymphocytes as shown in the gating strategy in Supplementary Figure S1. (SEM: Standard Error of Mean, SD: Standard Deviation, Min: Minimum, Max: Maximum).

**Table 2:** Summary EC_50_ (μg/ml) for 36 CD154 + T−cell subsets from 20 responders.

**T-cell Subsets**	**Median**	**Mean**	**SEM**	**Min**	**Max**	**SD**	**Test of Normality**					
							**Kolmogorov-Smirnov**			**Shapiro-Wilk**		
							**Statistic**	**df**	**p-value**	**Statistic**	**df**	**p-value**
ThM+28−154+	35.8	37.5	7.0	0.2	120.5	31.3	.148	20	.200*	.891	20	.029
TM+28−154+	30.1	40.2	9.7	0.0	177.9	43.2	.176	20	.105	.815	20	.001
Tc+154+	26.5	86.6	46.2	0.3	822.6	206.5	.424	15	.000	.413	15	.000
Tc+28+154+	22.7	22.2	3.9	0.4	52.7	17.5	.145	15	.200*	.926	15	.237
Tc+28−154+	21.0	40.7	10.6	0.3	128.1	47.3	.256	16	.006	.808	16	.003
Dn+154+	19.7	38.2	9.1	0.1	116.8	40.9	.208	18	.039	.828	18	.004
DnM+28+154+	16.3	37.0	10.1	0.5	151.9	45.2	.210	20	.021	.785	20	.001
TN+28−154+	15.1	18.9	3.6	0.2	48.9	15.9	.148	19	.200*	.905	19	.060
TcM+154+	14.6	62.8	32.9	0.2	623.1	146.9	.369	17	.000	.417	17	.000
ThM+154+	14.4	18.7	3.8	1.7	64.0	17.1	.173	20	.117	.838	20	.003
Th+154+	14.1	17.7	2.9	2.6	51.2	12.9	.167	20	.147	.911	20	.066
TM+CD154+	14.1	18.9	4.0	1.5	65.6	17.9	.185	20	.070	.840	20	.004
TN+CD154+	13.8	18.7	3.2	2.7	40.2	14.1	.240	20	.004	.850	20	.005
ThN+28+154+	13.7	18.3	3.2	1.7	49.2	14.5	.236	20	.005	.886	20	.023
Dn+28+154+	13.4	26.7	6.6	0.1	112.9	29.7	.206	20	.026	.818	20	.002
ThN+154+	13.2	17.9	3.0	1.9	52.3	13.2	.180	20	.088	.914	20	.076
TN+28+154+	12.9	17.9	3.0	2.4	43.5	13.5	.183	20	.078	.893	20	.031
T+154+	12.8	18.6	3.4	2.9	65.0	15.2	.174	20	.116	.847	20	.005
TcN+28+154+	12.5	22.9	6.5	0.4	105.4	29.5	.260	13	.016	.715	13	.001
Th+28+154+	12.4	16.4	2.7	2.0	43.7	12.1	.246	20	.003	.888	20	.024
T+28+154+	12.2	17.1	2.8	3.1	47.6	12.6	.182	20	.081	.896	20	.035
ThM+28+154+	12.1	16.7	3.4	1.0	59.7	15.1	.196	20	.043	.851	20	.006
TM+28+154+	12.0	15.6	3.1	0.7	59.0	13.9	.152	20	.200*	.845	20	.004
DnM+154+	11.9	32.2	10.9	0.0	144.1	48.8	.290	20	.000	.667	20	.000
Dn+28−154+	11.8	67.5	39.8	0.1	788.3	177.8	.381	19	.000	.388	19	.000
DnN+154+	11.7	21.5	5.3	0.1	65.0	23.5	.203	19	.038	.802	19	.001
T+28−154+	11.7	25.1	6.0	0.1	93.1	26.7	.241	20	.003	.842	20	.004
TcM+28−154+	11.3	26.3	7.8	0.2	105.7	34.8	.249	19	.003	.765	19	.000
Th+28−154+	11.1	28.9	9.2	1.0	174.5	41.1	.249	20	.002	.685	20	.000
DnN+28+154+	10.7	19.1	4.9	0.2	81.8	22.0	.242	20	.003	.797	20	.001
TcM+28+154+	10.4	19.2	4.7	0.2	73.8	21.0	.202	18	.050	.835	18	.005
ThN+28−154+	9.4	51.8	33.3	0.4	679.7	148.9	.416	20	.000	.334	20	.000
TcN+154+	9.1	16.7	4.6	0.0	66.0	20.7	.224	16	.031	.792	16	.002
DnM+28−154+	6.6	31.9	10.3	0.0	133.9	46.2	.287	18	.000	.723	18	.000
TcN+28−154+	6.5	14.7	5.2	0.2	100.5	23.9	.282	18	.001	.579	18	.000
DnN+28−154+	3.2	36.8	17.1	0.0	302.0	76.5	.315	16	.000	.540	16	.000

Also shown are statistical tests of normality for EC_50_ distribution in each subset. The EC_50_ for the majority of subsets shows significant p-values from the two tests of normality and therefore does not follow normal distribution. (SEM: Standard Error of Mean, SD: Standard Deviation, Min: Minimum, Max: Maximum).

**Table 3:** Variation in frequency and EC_50_ for two candidate T-cells subsets with the highest EC_50_.

		**Median**	**Mean**	**SD**	**Min**	**Max**	**%CV**	**CI**
ThM28-	Frequency	0.17	1.24	2.40	0.02	9.91	193.5	0.38–2.11
	EC_50_	35.8	37.5	31.3	0.2	120.5	83.5	22.9–52.1
Tc	Frequency	29.97	29.04	8.53	7.37	45.87	29.4	25.97–32.11
	EC_50_	26.5	86.6	206.5	0.3	822.6	238.5	−17.8–200.9

The measure of variation is the coefficient of variation or CV (n = 20 responders).

**Table 4:** General demographics and clinical information for four Rejectors (R) and seven Non-rejectors (NR).

	**R**	**NR**
Total subjects	4	7
Time from Transplant to Sample collection (days)	2778 ± 1160	1162 ± 419
Age on day of sample collection (Years)	16.3 ± 4.7	4.6 ± 1.3
Gender (M:F)	3:1	4:3
Race (Caucasian: Other)	3:1	7:0
LTx:LKTx:ITx:LITx	3:0:1:0	5:1:0:1
Tacrolimus whole blood concentrations	7.4 ± 1.9	5.2 ± 1.0

All four Rejectors used in this study experienced biopsy proven acute cellular rejection on the day of blood sampling. LTx: Liver transplant, LKTx: combined liver kidney transplant, ITx: Intestine transplant, and LITx: combined liver intestine transplant.
