# Supplementary material for: Alloreactive CD154-expressing T-cell subsets with differential sensitivity to the immunosuppressant, belatacept: potential targets of novel belatacept-based regimens
Source: Sci Rep. 2015 Oct 16;5:15218. doi: 10.1038/srep15218 (PMC4607954; doi:10.1038/srep15218)
Supplement: Supplementary Information [file srep15218-s2.pdf]

Supplementary Information

Alloreactive CD154-expressing T-cell subsets with differential sensitivity to the immunosuppressant, belatacept: potential targets of novel belatacept-based regimens

Chethan Ashokkumar[1,3], Bishu Ganguly[2], Robert Townsend[2], Jaimie White[1], Samantha Levy[1], Michael Moritz[3], George Mazariegos[3], Qing Sun[3], & Rakesh Sindhi[3*]

[1]Plexision, Inc., 4424 Penn Avenue, #202, Pittsburgh, PA 15224 USA

Chethan.ashokkumar@plexision.com; Jaimie.white@plexision.com; Samantha.levy@plexision.com

[2]Bristol-Myers-Squibb, PO Box 4000, Princeton, NJ 08543-4000 USA

Bishu.Ganguly@bms.com; robert.townsend@bms.com

[3]Children's Hospital of Pittsburgh of University of Pittsburgh Medical Center, 4401 Penn Avenue, Pittsburgh, PA 15224. USA

Chethan.ashokkumar@chp.edu; qing.sun@chp.edu; Michael.Moritz@chp.edu ;

George. Mazariegos@chp.edu; *rakesh.sindhi@chp.edu (412-692-7123)

Supplementary Fig S1

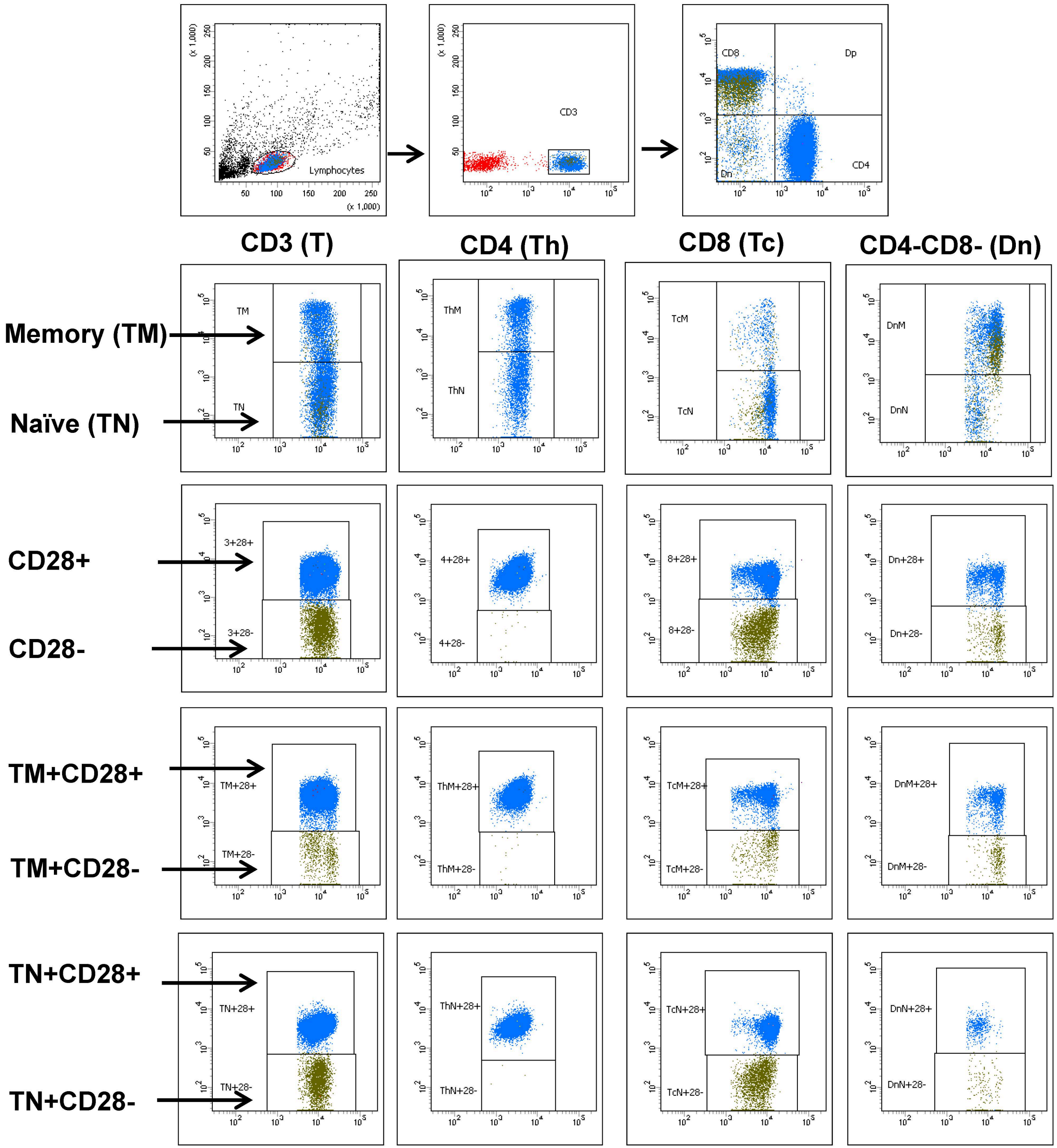

Supplementary Fig S2

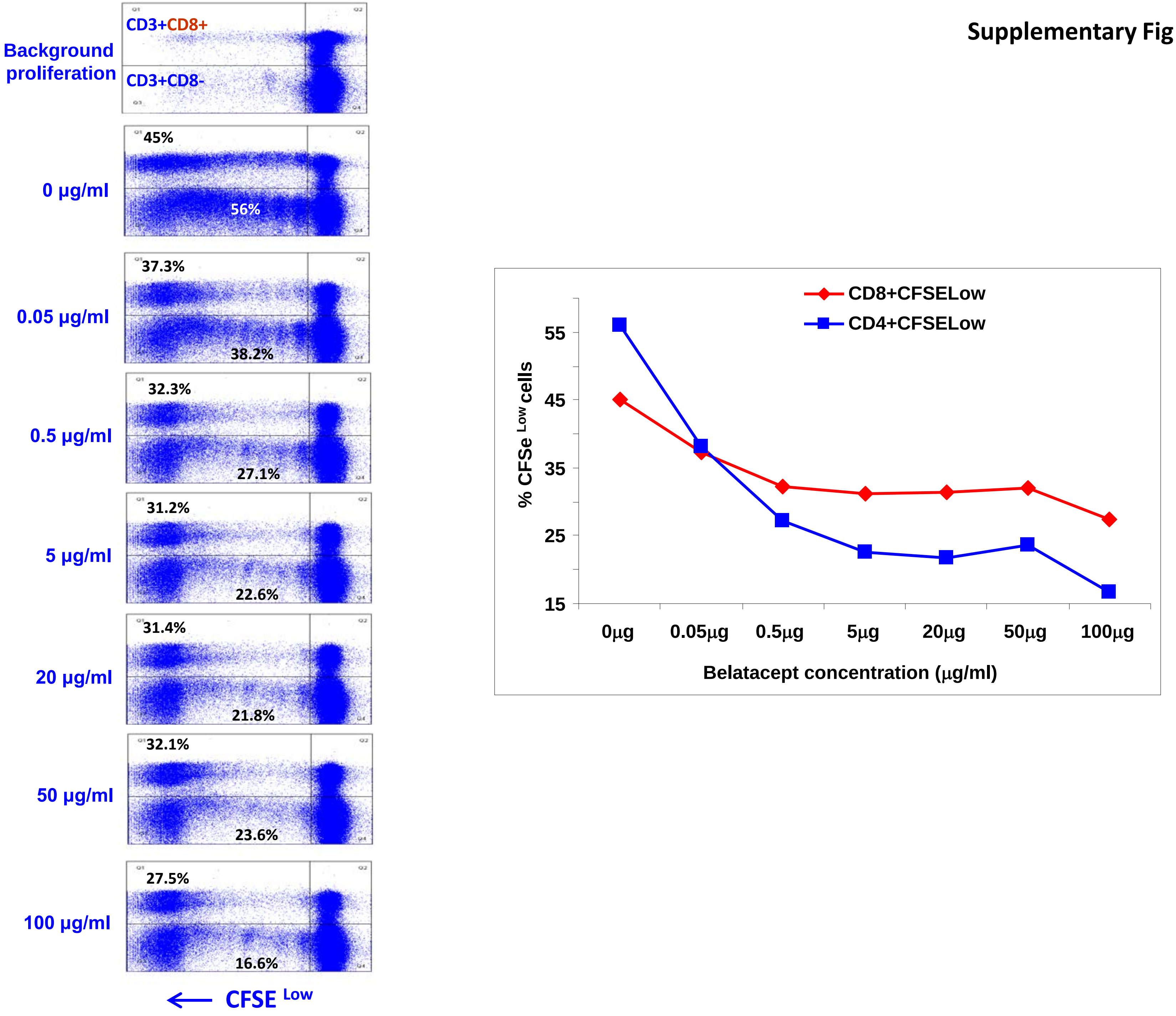

Supplementary Fig S3

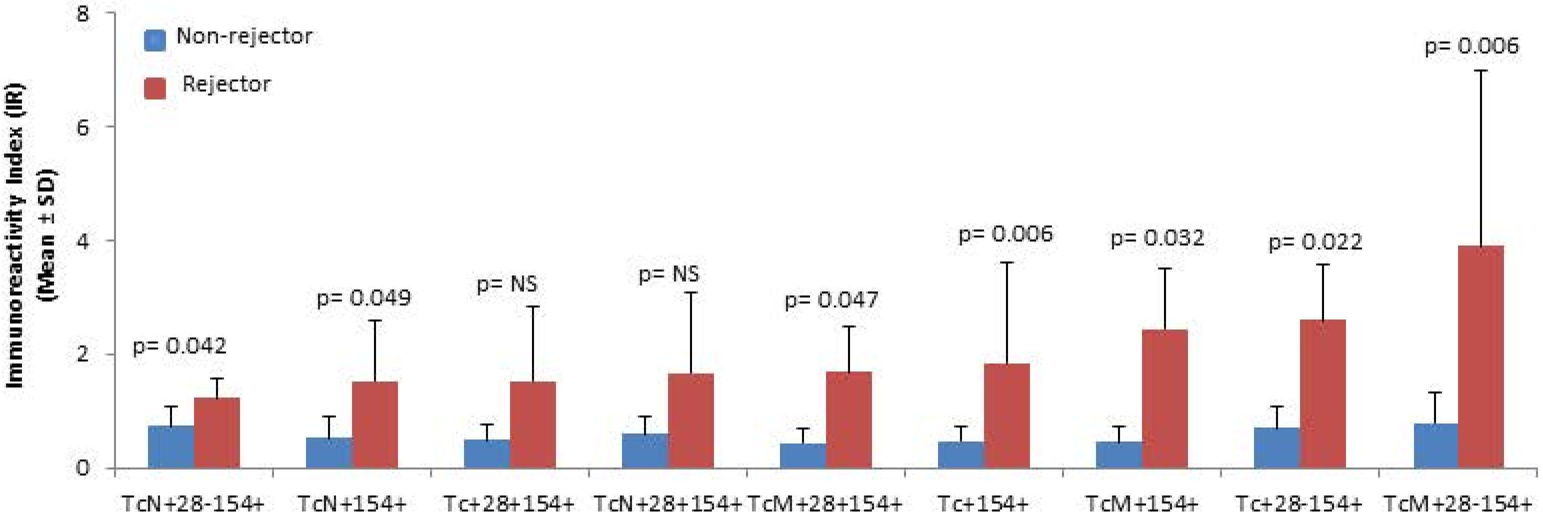

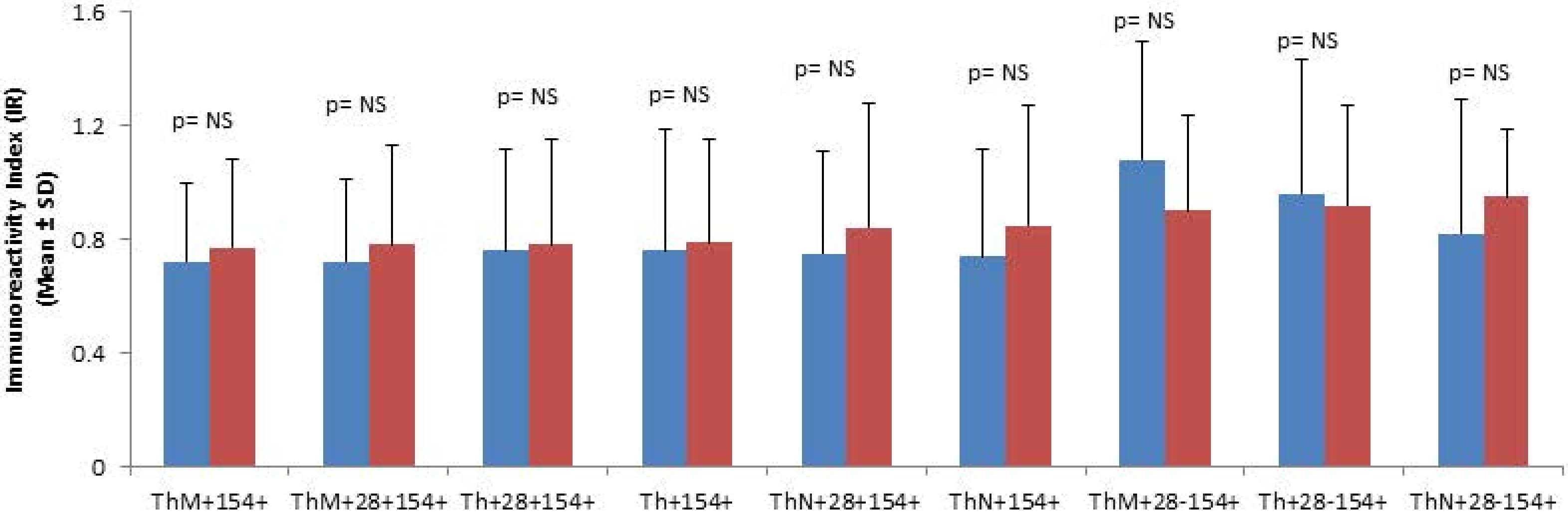

Supplementary Fig S4

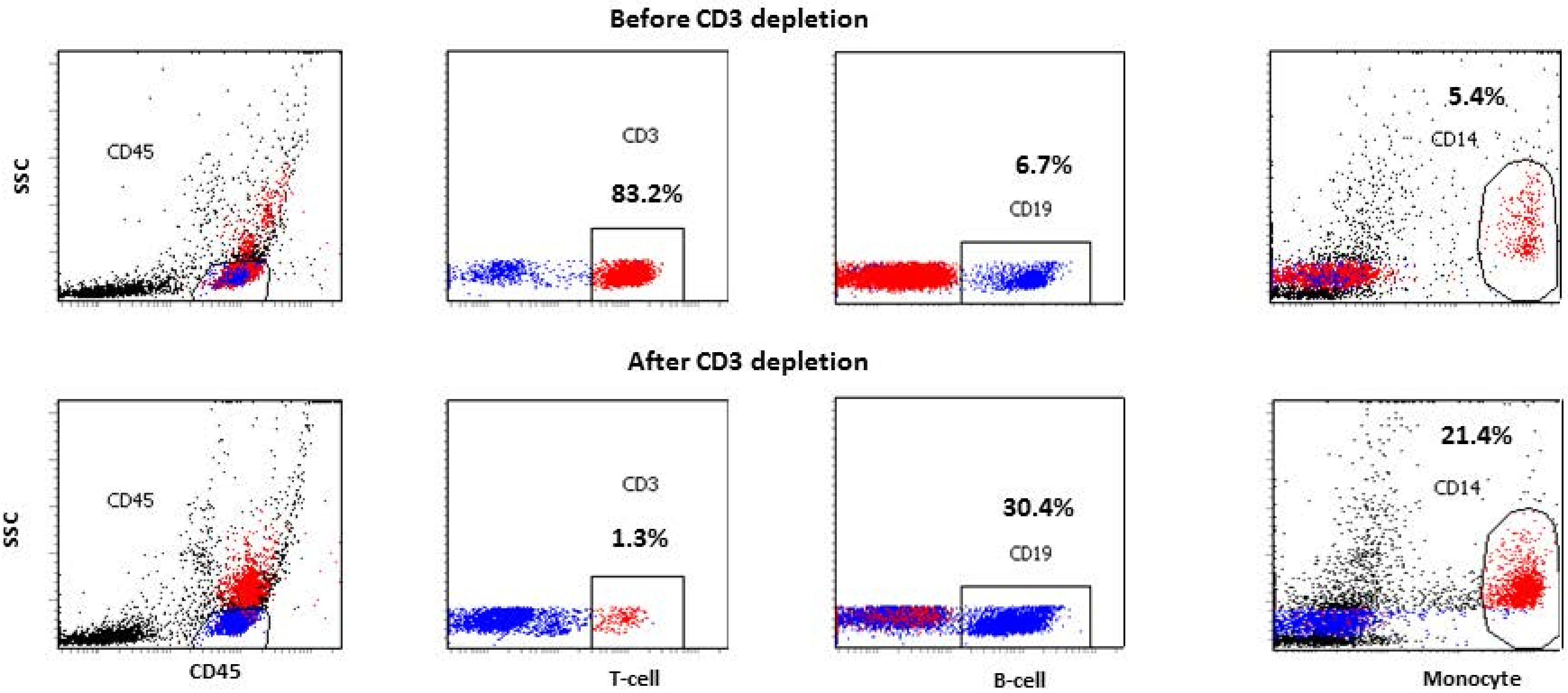

Supplementary Table S2:

Subset	Mean (IR)		Median (IR)		S.D		Minimum		Maximum		
	NR	R	NR	R	NR	R	NR	R	NR	R	p-value
TcM+28+154+	0.44	1.69	0.42	1.73	0.24	0.79	0.09	0.70	0.85	2.58	**0.047**
TcM+28-154+	0.78	3.90	0.55	2.64	0.54	3.10	0.24	1.83	1.79	8.50	**0.006***
TcM+154+	0.45	2.44	0.47	2.38	0.27	1.08	0.09	1.33	0.94	3.67	**0.032**
TcN+28+154+	0.60	1.67	0.71	1.25	0.31	1.43	0.19	0.47	1.000	3.71	0.229 (NS)
TcN+28-154+	0.73	1.23	0.57	1.21	0.36	0.35	0.43	0.89	1.50	1.62	**0.042***
TcN+154+	0.53	1.52	0.500	1.33	0.37	1.08	0.08	0.41	1.00	3.00	**0.049**
Tc+28+154+	0.48	1.53	0.57	1.07	0.27	1.29	0.09	0.57	0.79	3.43	0.2(NS)
Tc+28-154+	0.70	2.59	0.57	2.79	0.36	0.97	0.18	1.50	1.29	3.67	**0.022**
Tc+154+	0.46	1.84	0.50	1.00	0.28	1.78	0.07	0.86	0.75	4.50	**0.006***
DnM+28+154+	1.22	1.23	0.72	0.76	1.57	1.29	0.11	0.31	0.47	3.10	0.999* (NS)
DnM+28-154+	1.26	1.32	0.77	0.76	1.19	1.3	0.61	0.53	3.92	3.25	0.527* (NS)
DnM+154+	1.75	1.62	0.71	0.83	2.94	1.97	0.12	0.27	8.38	4.53	0.999* (NS)
DnN+28+154+	0.91	1.03	0.83	1.03	0.46	0.54	0.41	0.48	1.63	1.57	0.71 (NS)
DnN+28-154+	0.90	1.19	0.79	1.06	0.76	0.47	0.1	0.76	2.37	1.86	0.518 (NS)
DnN+154+	0.93	1.60	0.84	0.97	0.58	1.83	0.32	0.25	1.76	4.21	0.382 (NS)
Dn+28+154+	1.03	1.12	0.73	0.87	0.93	1.01	0.27	0.28	2.96	2.48	0.927* (NS)
Dn+28-154+	2.23	2.20	0.73	0.84	4.36	3.17	0.12	0.20	12.09	6.93	0.648* (NS)
DOUBNEG+154+	1.30	1.85	0.78	1.39	1.69	1.98	0.21	0.21	5.03	4.43	0.999* (NS)
ThM+28+154+	0.72	0.78	0.65	0.88	0.29	0.35	0.33	0.27	1.13	1.07	0.782 (NS)
ThM+28-154+	1.08	0.90	1.05	0.92	0.42	0.34	0.51	0.51	1.69	1.25	0.488 (NS)
ThM+154+	0.72	0.77	0.65	0.88	0.28	0.31	0.36	0.32	1.13	1.01	0.772 (NS)
ThN+28+154+	0.75	0.84	0.52	0.94	0.36	0.44	0.43	0.22	1.42	1.24	0.733 (NS)
ThN+28-154+	0.82	0.95	0.76	0.95	0.47	0.24	0.32	0.66	1.79	1.23	0.631 (NS)
ThN+154+	0.74	0.85	0.52	0.95	0.38	0.42	0.41	0.27	1.45	1.25	0.674 (NS)
Th+28+154+	0.76	0.78	0.55	0.90	0.36	0.37	0.47	0.25	1.44	1.07	0.927 (NS)
Th+28-154+	0.96	0.92	0.68	0.86	0.47	0.35	0.52	0.61	1.76	1.35	0.886 (NS)
Th+154+	0.76	0.79	0.55	0.89	0.43	0.36	0.43	0.29	1.62	1.08	0.788* (NS)
TM+28+154+	0.84	0.78	1.01	0.85	0.29	0.32	0.37	0.36	1.06	1.06	0.648* (NS)
TM+28-154+	2.63	1.58	0.73	1.42	5.11	1.31	0.30	0.22	14.18	3.28	0.788* (NS)
TM+CD154+	0.87	0.95	1.00	0.85	0.34	0.58	0.33	0.39	1.18	1.71	0.999* (NS)
TN+28+154+	0.81	0.82	0.68	0.81	0.34	0.35	0.45	0.43	1.30	1.25	0.947 (NS)
TN+28-154+	0.83	1.60	1.80	1.70	0.39	0.97	0.42	0.42	1.45	2.58	0.087 (NS)
TN+CD154+	0.83	0.92	0.94	0.83	0.29	0.46	0.52	1.49	1.29	1.49	0.704 (NS)
T+28+154+	0.79	0.82	0.74	0.89	0.32	0.38	0.43	0.32	1.31	1.17	0.89 (NS)
T+28-154+	1.77	1.65	0.65	1.41	2.74	1.31	0.49	0.36	7.93	3.44	0.648* (NS)
T+154+	0.82	1.01	0.92	0.91	0.37	0.64	0.38	0.38	1.46	1.85	0.529 (NS)

Supplementary Figure S1. Gating strategy to divide T-cells into 36 subsets by flow cytometry. Upper row of scatterplots shows that CD3 cells are selected from the lymphocyte gate and divided into CD3 and CD8 cells. Double-negative T-cells are in the upper right quadrant of the last scatterplot in the upper row. Below this upper row are four rows of scatterplots. Each row shows the derivation of the subsets from the four major T-cells subsets. These major subsets are T-cell (CD3), T-helper (CD4), T-cytotoxic (Tc, CD8) and double negative (Dn, CD4-CD8-) T-cell subsets. Each major subset is divided into memory (M) and naïve (N), CD28-expressing or CD28+, and CD28-negative (CD28-) subsets. The memory and naïve subsets are each further divided into CD28+ and CD28- subsets. In this way, each major subset contributes nine subsets to the overall T-cell subpopulation for a total of 36 T-cell subsets.

Supplementary Figure S2. (Left) Scatterplots show effect of belatacept on proliferative alloresponse measured by CFSE-dye dilution in 7-day co-culture of responder and stimulator in 1: 2 ratio. (Right) Panel shows belatacept-concentration-dependent inhibition of Th and Tc from a responder.

Supplementary Figure S3. Bar diagrams show mean and standard deviation (error bars) of IR values for rejectors and non-rejectors for nine subsets of the Tc (upper panel) and Th (lower panel) parent subsets.

Supplementary Figure S4. Scatterplots in the upper panel show frequencies of CD45+ cells which are CD3+T-cells and CD19+B-cells. Also shown are frequencies of CD14+monocytes. These frequencies are seen in undepleted stimulator cells from a normal human subject. Scatterplots in the lower panel are obtained from PBL after T-cell depletion, and show depletion of CD3+T-cells to <2% of the CD45+ population. Frequencies of CD19+B-cells and CD14+monocytes show enrichment by five- and four-fold, respectively, over frequencies seen in undepleted PBL.

Supplementary Table S1. Upper table shows Spearman rho values for correlations between EC_{50} and frequencies for 36 subsets from 20 responders. Lower table shows the p-values for the respective Spearman correlations.

Supplementary Table S2: Summary immunoreactivity index values for research blood samples from four rejectors (R) and 7 non-rejectors (NR) with liver (n=8), intestine (n=1), combined liver-kidney (n=1, and combined liver-intestine (n=1) transplants. The IR value is the multiple by which donor-induced CD154+T-cells within any subset exceed those induced by HLA-non-identical or reference cells. Rejectors and non-rejectors are compared with t-tests (normal distribution) or with Mann-Whitney test (*) if values are not normally distributed. P-values marked with an asterisk (*) are from the Mann-Whitney test.
